# Surgeon-designed patient-specific instrumentation improves glenoid component screw placement for reverse total shoulder arthroplasty in a population with small glenoid dimensions

**DOI:** 10.1007/s00264-023-05706-z

**Published:** 2023-02-10

**Authors:** Colin Shing-Yat Yung, Christian Fang, Evan Fang, Yuk-Chuen Siu, Dennis King Hang Yee, Kevin Kwun-Hung Wong, Kai-Chung Poon, Matthew Man Fai Leung, Jonathan Wan, Tak-Wing Lau, Frankie Leung

**Affiliations:** 1 Department of Orthopaedics and Traumatology, The University of Hong Kong, 5/F Professional Block, Queen Mary Hospital, 102 Pokfulam Road, Pokfulam, Hong Kong; 2 Department of Orthopaedics and Traumatology, North District Hospital, Sheung Shui, Hong Kong; 3 Department of Orthopaedics and Traumatology, Alice Ho Miu Ling Nethersole Hospital, Tai Po, Hong Kong; 4 Department of Orthopaedics and Traumatology, Kwong Wah Hospital, Yau Ma Tei, Hong Kong; 5 Department of Prosthetics and Orthotics, Pamela Youde Nethersole Eastern Hospital, Chai Wan, Hong Kong

**Keywords:** Reverse total shoulder arthroplasty, Reverse shoulder replacement, 3D printing, Glenosphere, Custom surgical template, Screw length

## Abstract

**Purpose:**

Glenoid component loosening is a potential complication of reverse total shoulder arthroplasty (rTSA), occurring in part due to lack of adequate screw purchase in quality scapular bone stock. This study was to determine the efficacy of a surgeon-designed, 3D-printed patient-specific instrumentation (PSI) compared to conventional instrumentation (CI) in achieving longer superior and inferior screw lengths for glenoid component fixation.

**Methods:**

A multi-centre retrospective analysis of patients who underwent rTSA between 2015 and 2020. Lengths of the superior and inferior locking screws inserted for fixation of the glenoid baseplate component were recorded and compared according to whether patients received PSI or CI. Secondary outcomes included operative duration and incidence of complications requiring revision surgery.

**Results:**

Seventy-three patients (31 PSI vs. 42 CI) were analysed. Average glenoid diameter was 24.5 mm (SD: 3.1) and 81% of patients had smaller glenoid dimensions compared to the baseplate itself. PSI produced significantly longer superior (44.7 vs. 30.7 mm; *P* < 0.001) and inferior (43.0 vs. 31 mm; *P* < 0.001) mean screw lengths, as compared to CI. A greater proportion of maximal screw lengths for the given rTSA construct (48 mm) were observed in the PSI group (71.9% vs. 11.9% superior, 59.4% vs. 11.9% inferior). Operative duration was not statistically significantly different between the PSI and CI groups (150 min vs. 169 min, respectively; *P* = 0.229). No patients had radiographic loosening of the glenoid component with an average of 2-year follow-up.

**Conclusion:**

PSI facilitates longer superior and inferior screw placement in the fixation of the glenoid component for rTSA. With sufficient training, PSI can be designed and implemented by surgeons themselves.

## Introduction

Reverse shoulder arthroplasty (rTSA) is indicated for a number of conditions, including rotator cuff tear arthropathy, glenohumeral arthritis, and three- and four-part proximal humerus fractures in the elderly [1]. rTSA has demonstrated better outcomes and fewer complications, as compared to hemiarthroplasty or plate fixation for severe proximal humerus fractures [2–5]. However, complications of rTSA remain frequent and are diverse in nature [1]. Failure of the glenoid component fixation is an important complication arising from rTSA, occurring due to component malpositioning and lack of implant purchase in adequate scapular bone stock [6, 7]. Longer glenoid component screws have shown to improve initial fixation stability and reduce micromotion [7–9]. As such, achieving better screw purchase by maximising screw length may improve implant longevity. This is particularly challenging in the Asian population with previous studies demonstrating smaller glenoid dimensions and scapular geometry [10, 11]. The average glenoid diameter in a study from Korea was notable smaller than the component baseplate itself (27 mm) [11]. Similarly, females in Japan had an average glenoid diameter of only 23.4 mm [10]. Inherently small scapular bone in the Asian population makes accurate screw insertion for two large 5 mm diameter screws, increasingly problematic. There is also a wide variation in scapular bone morphology and density over short distances and no means of identifying regions of high quality bone stock intra-operatively [8]. Previously, intra-operative computer navigation has been used to address these issues, but inherent technical drawbacks and lengthened operating time preclude its popularisation [12].

Patient-specific instrumentation (PSI) using three-dimensionally (3D) printed guides is an increasingly popular tool for improving surgical accuracy across various contexts. PSI has been shown to produce more accurate glenoid component placement in rTSA and total shoulder arthroplasty [6, 12, 13]. However, the efficacy of PSI inachieving better screw purchase in scapular bone has not been evaluated before. Of particular interest are the superior and inferior locking screws of the glenoid component due to their collinearity with the direction of load transmission [7]. Since 2018, our centres have adopted a surgeon-designed PSI workflow for rTSA glenoid component placement using PSI guides manufactured at a central printing laboratory. The objective of this study was to determine the efficacy of surgeon-designed PSI for superior and inferior screw placement in glenoid component fixation. Our hypothesis is that surgeon-designed PSI enables longer superior and inferior screw lengths as compared to conventional instrumentation (CI).

## Materials and methods

### Study design and setting

This was a retrospective, multi-centre comparative study conducted using data from eight hospitals. Ethical approval was sought and satisfied (HKU/HA HKW reference no. UW 21–111). Consecutive patients who had undergone rTSA using the Delta Xtend Reverse Shoulder System (DePuy Synthes, Raynham, MA, USA) between 2015 and 2020 were recruited. The inclusion criteria were age 50 years or older, indicated for rTSA for a non-reconstructable proximal humerus fracture, glenohumeral arthritis, or rotator cuff arthropathy with good premorbid function. Patients were excluded if they had axillary nerve deficit, active infection, local neoplastic conditions, or severe bone loss leading to the need for customised implants or two-stage reconstruction procedures. Historical control patients operated before 2018 by the same surgeons had CI performed prior to implementation of 3D-printed PSI. After 2018, both PSI and CI were used depending on centre and surgeon preference.

### Data collection and analysis

**Fig. 1:**
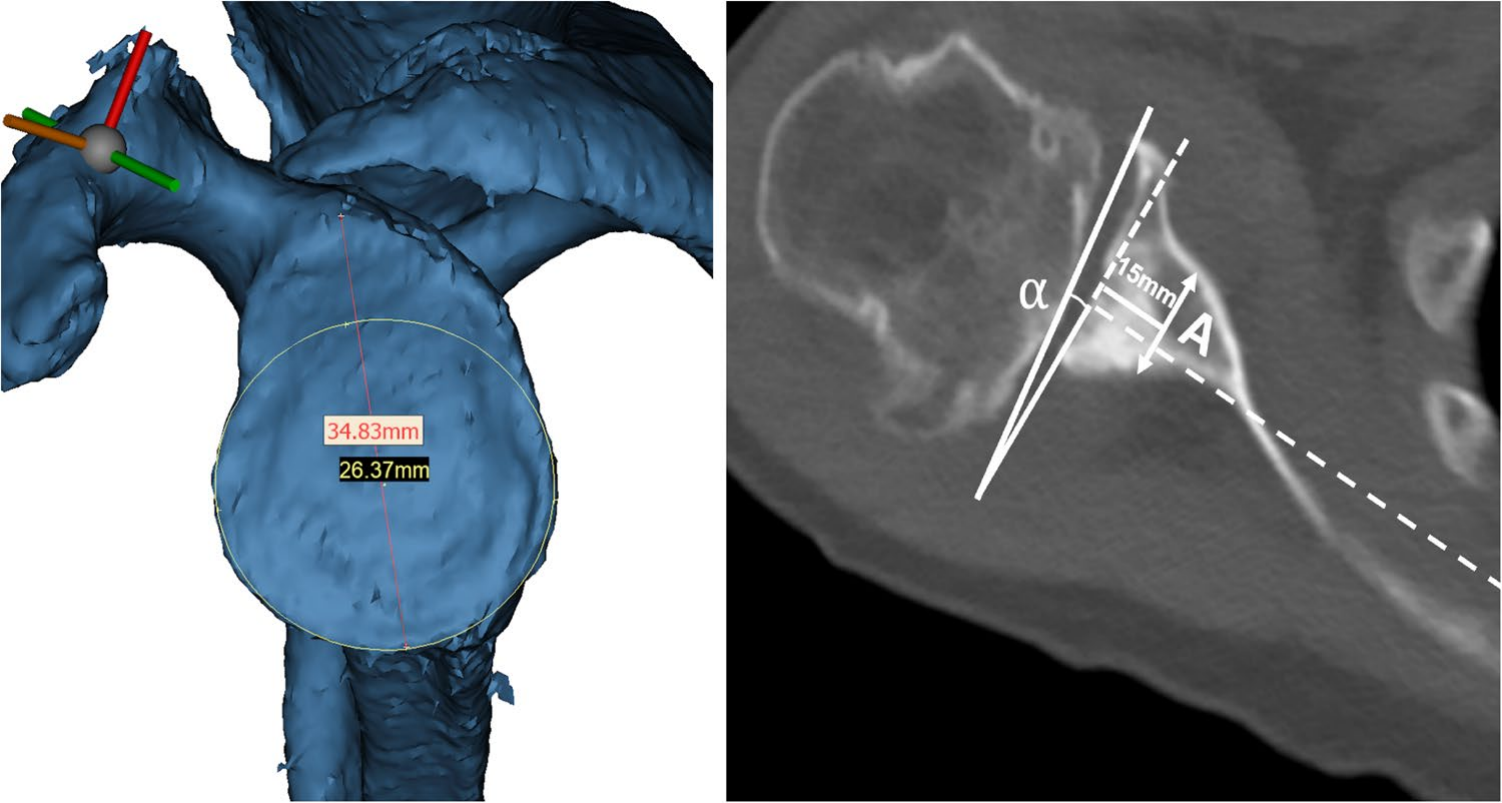
Glenoid AP distance (diameter) and glenoid height measured in en-face view (right). Scapular neck AP distance and glenoid version measured in axial view. Distance A is the glenoid neck AP dimension measured 15 mm proximally to the articular surface, perpendicular to the scapular axis. Angle α is the angle subtended by the plane of the glenoid articular surface and the line perpendicular to the scapular axis (left)

Patient demographics, operative details, and computed tomography (CT) scans were retrieved from a centralised patient database. Pre-operative glenoid dimensions were measured for comparison and assessment of the difficulty for screw insertion. Using a 3D reconstruction of the glenoid in en-face view, the glenoid anterior–posterior (AP) dimension was measured as the diameter of the best-fitting circle for the glenoid centre; glenoid height was measured as the distance between the highest and lowest points on the low axis of the glenoid cavity (Fig. 1) [11]. Using a 2D axial view of the scapula, the scapular neck AP dimension was measured as the width of the scapula perpendicular to the scapular axis, 15 mm proximal to the glenoid rim (Fig. 1) [10]. Glenoid version was measured as the angle made between the plane of the articular surface of the glenoid and the scapular axis.

Baseline characteristics were compared between groups. The superior and inferior glenoid component screw lengths were recorded. Secondary outcomes included operative duration and incidence of complications requiring revision surgery. Statistical analysis was performed using SPSS Statistics version 26 (IBM, Armonk, NY, USA). Patients were analysed according to whether they received PSI or CI during operation. Data were analysed using independent samples *t-*tests for continuous variables and Fisher’s exact tests for categorical variables. A two-tailed *P*-value of < 0.05 was considered statistically significant.

### PSI jig design and fabrication

**Fig. 2:**
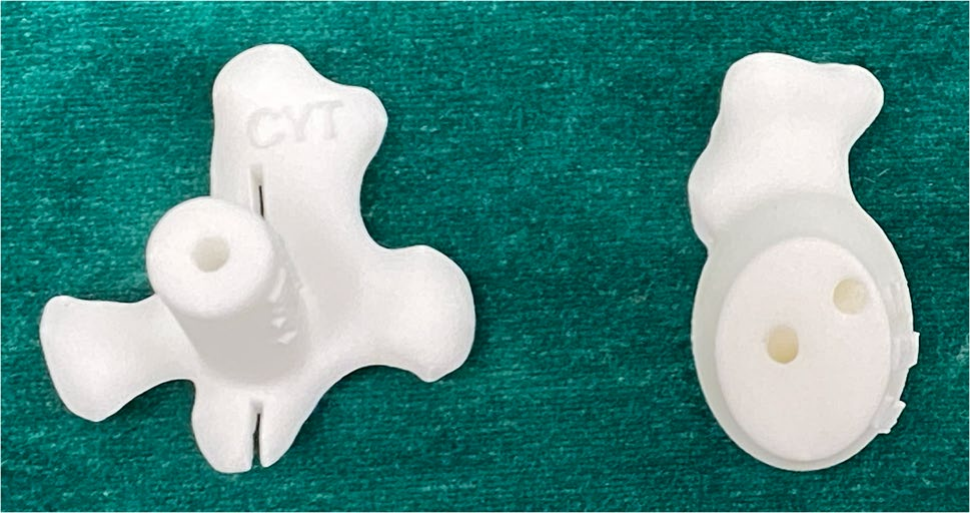
PSI central metaglene peg (left) and screw guide (right) components

The PSI jigs consisted of two components: a guide for the central metaglene peg placement and a guide for the superior and inferior locking screws (Fig. 2). All jigs were designed and verified by the surgeon author CF using Meshmixer (Autodesk, San Rafael, CA, USA) and 3-Matics software (Materialise, Leuven, Belgium). The jigs were created according to four design principles: (1) priority was given to retaining glenoid bone stock by minimising the need for reaming without additional inferior tilting; (2) the native glenoid version angle was followed unless it deviated from the scapular body plane by greater than 20°; (3) the roll of the metaglene was determined by optimising the superior and inferior screw trajectories; and (4) the inferior margin of the metaglene remained flush with the inferior margin of the native glenoid to avoid subsequent impingement and notching. The step-by-step design methodology can be viewed at Supplementary Material: https://www.youtube.com/watch?v=bwGJMU_j_WI.

**Fig. 3:**
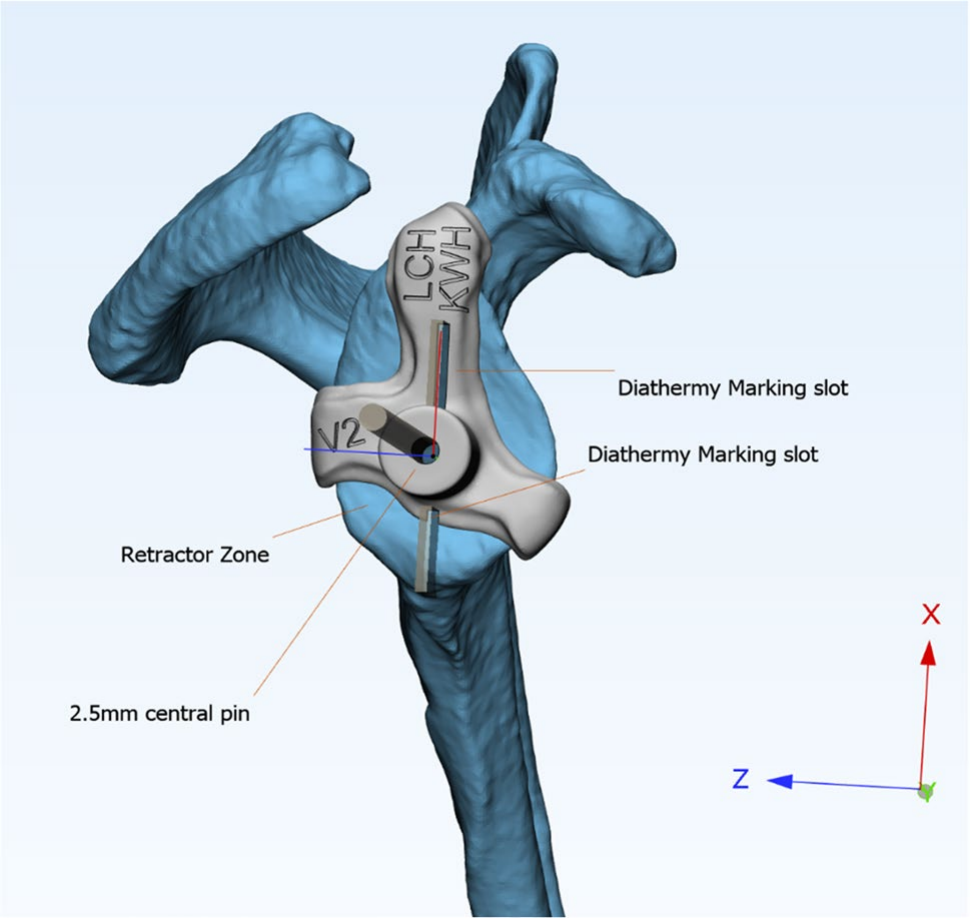
3D model of the metaglene peg guide with coracoid base and anterior–posterior glenoid rim referencing. Distance between the three reference points is maximised for stable guide placement

**Fig. 4:**
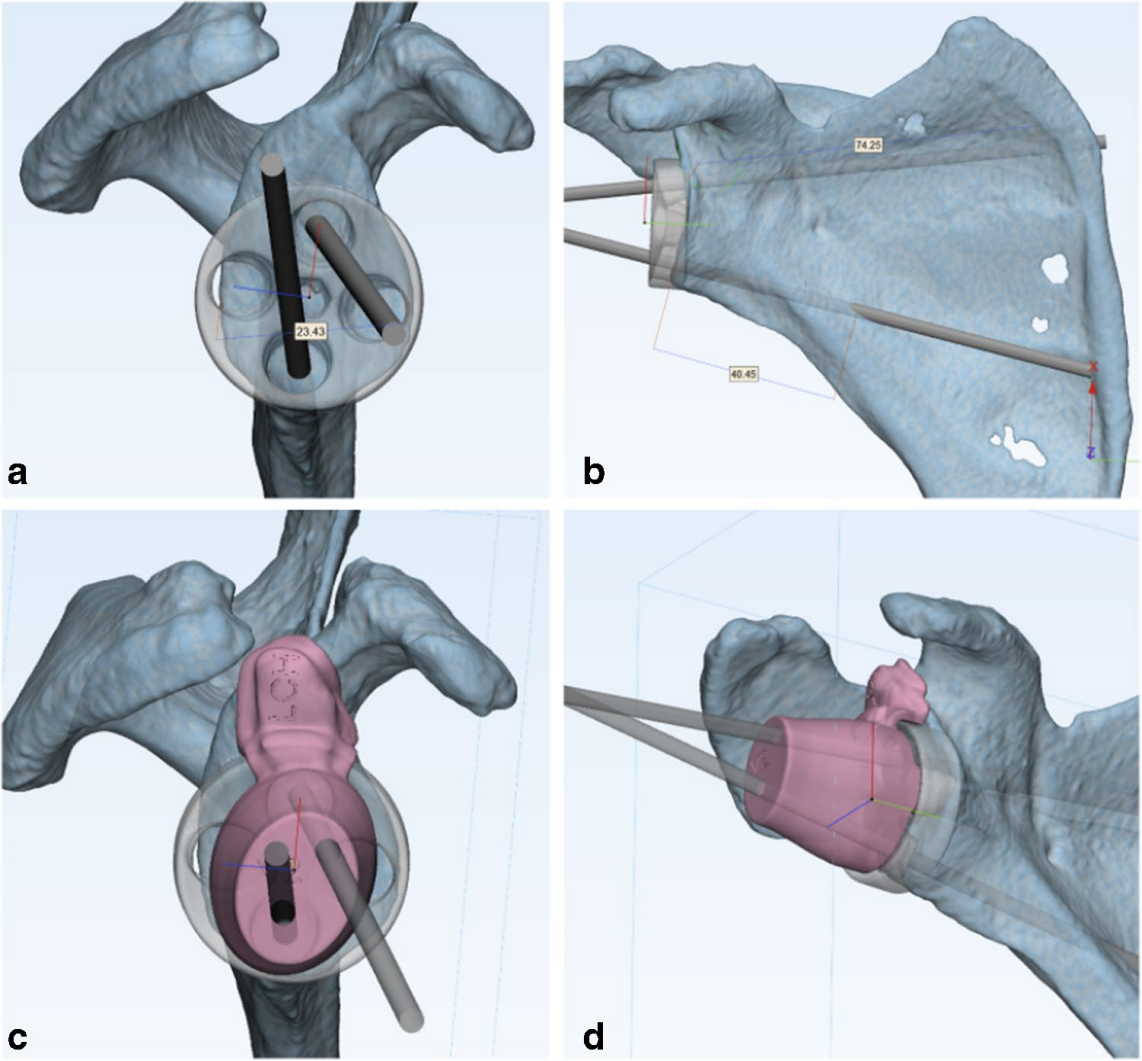
**a**–**b** 3D model of metaglene with planned screw trajectories. **c**–**d** Screw guide component fitted over metaglene

CT scans were obtained in Digital Imaging and Communications in Medicine (DICOM) format with 1.0 mm or finer slice thickness and segmented using Mimics v.21 software (Materialise, Leuven, Belgium). The central peg guide was first created using the coracoid base and the anteroinferior and posterior glenoid rim as surface reference points (Fig. 3). Two diathermy marking slots were included for vertical axis referencing. The markings help orientate the centre of the superior and inferior screw holes as well as the depth of glenoid cartilage for guiding reaming. Next, a model of the planned metaglene was created and placed over the glenoid model as a reference for the screw guide. The optimal superior and inferior screw trajectories were determined by placing cylinders with diameters corresponding to the planned screws through the 3D scapula model. The screw trajectories were placed such that the screw length was maximised (Fig. 4). These cylinders were then subtracted from the screw guide model to create the final screw guide, which included protrusions to fit into the metaglene screw holes as well as a reference area that fit over the coracoid base. The jigs were 3D printed using medically designated acrylonitrile butadiene styrene (ABS-M30i, Stratasys, MI, USA) or nylon 12 (PA2200, EOS, Krailling, Germany) by either an industrial-grade fused deposition modelling (FDM) printer (Fortus 450 mc, Stratasys, MI, USA) or selective laser sintering (SLS) printer (Formiga P110, EOS, Krailling, Germany). Each screw guide was tested pre-operatively by fitting onto the metaglene with the drill bit inserted.

### Surgical method

In the conventional method, the operational procedure follows the manufacturer’s specifications. In the PSI method, the glenoid is exposed with circumferential release of glenoid and coracoid base soft tissue to ensure correct jig placement. The central peg jig is fitted to the bony anatomy of the coracoid base. A diathermy burn mark is made through the full depth of the articular cartilage along the vertical axis. The burn mark is then reamed until barely visible to ensure proper jig seating and additional marking on the subchondral bone is made as needed. The metaglene is implanted, and superior and inferior drilling and screw placement is performed using the screw jig. The lengths of the two screws are determined using a standard depth gauge inserted to the far cortex. Screw lengths are available in increments of 6 mm, and screw lengths in between sizes will be rounded to the nearest number or rounded up if in the middle. Anterior and posterior screws are placed only if the surgeon feels that there is inadequate purchase of the initial two screws.

## Results

A total of 73 patients (6 male, 67 female) were included in the analysis, of whom 31 received PSI and 42 received CI (Table 1). Mean age was 76.4 years (SD: 8.5; range: 54–93 years). Proximal humerus fracture was the most common indication for rTSA, accounting for 48 patients (66%). Glenoid geometry measurements confirmed uniformly small glenoid dimensions in our patient population group with the average glenoid AP diameter of 24.5 mm (SD: 3.1) and scapular neck length of only 10.1 mm (SD: 1.8). The small scapular geometry is emphasised with 59 (81%) patients having a glenoid diameter smaller than the designed baseplate itself (27 mm) as show in the example in Fig. 4. The mean turnover time for PSI jig production, from pre-operative CT to operation date, was 22.5 days (range: 1–226 days). The large range was affected by one outlier with patient operation deferred due to patient’s medical fitness. Mean follow-up time was 3.5 years (SD: 1.7) in the CI group and 2.1 years (SD: 1.1) in the PSI group.

**Table 1:** Patient characteristics

Characteristics	Conventional instrumentation (*n* = 42)	Patient-specific instrumentation (*n* = 31)	*P*-value	All patients (*n* = 73)
Sex				
Male	3 (7.1)	3 (9.7)	1.000	6 (8.2)
Female	39 (92.9)	28 (90.3)	1.000	67 (91.8)
Age (years)	76.8 ± 8.2	75.8 ± 8.9	.633	76.4 ± 8.5
Body weight (kg)	59.6 ± 15.6	59.3 ± 12.9	.933	59.4 ± 14.0
Height (cm)	153.5 ± 5.3	153.5 ± 7.7	1.000	153.5 ± 6.4
BMI (kg/m^2^)	26.0 ± 6.2	27.3 ± 4.4	.483	26.6 ± 5.4
Glenoid height (mm)	34.4 ± 2.8	36.8 ± 3.5	**.007**	35.7 ± 3.4
Glenoid AP (mm)	23.5 ± 2.0	25.8 ± 3.8	**.004**	24.5 ± 3.1
Glenoid AP less than 27 mm	37 (88.1%)	22 (70.9%)	.079	59 (80.8%)
Scapular neck AP (mm)	10.0 ± 1.7	10.1 ± 1.9	.829	10.1 ± 1.8
Glenoid version (degrees)	+ 2.7 ± 4.3	− 0.35 ± 5.9	**.029**	1.4 ± 5.3
Glenoid version beyond + / − 10 degrees	1 (2.4)	0	1.000	1 (1.4)
Indication for rTSA				
Proximal humerus fracture	33	16	**.015**	49
Post-traumatic arthropathy	6	8	.217	14
Rotator cuff arthropathy	1	3	.305	4
Other shoulder arthritis	2	4	.391	6

Data are either mean ± SD or *n *(%). Statistically significant value *p* < 0.05

Abbreviations: *AP* = anterior-posterior; *AVN* = avascular necrosis; *OA* = osteoarthritis; *rTSA* = reverse total shoulder arthroplasty

**Fig. 5:**
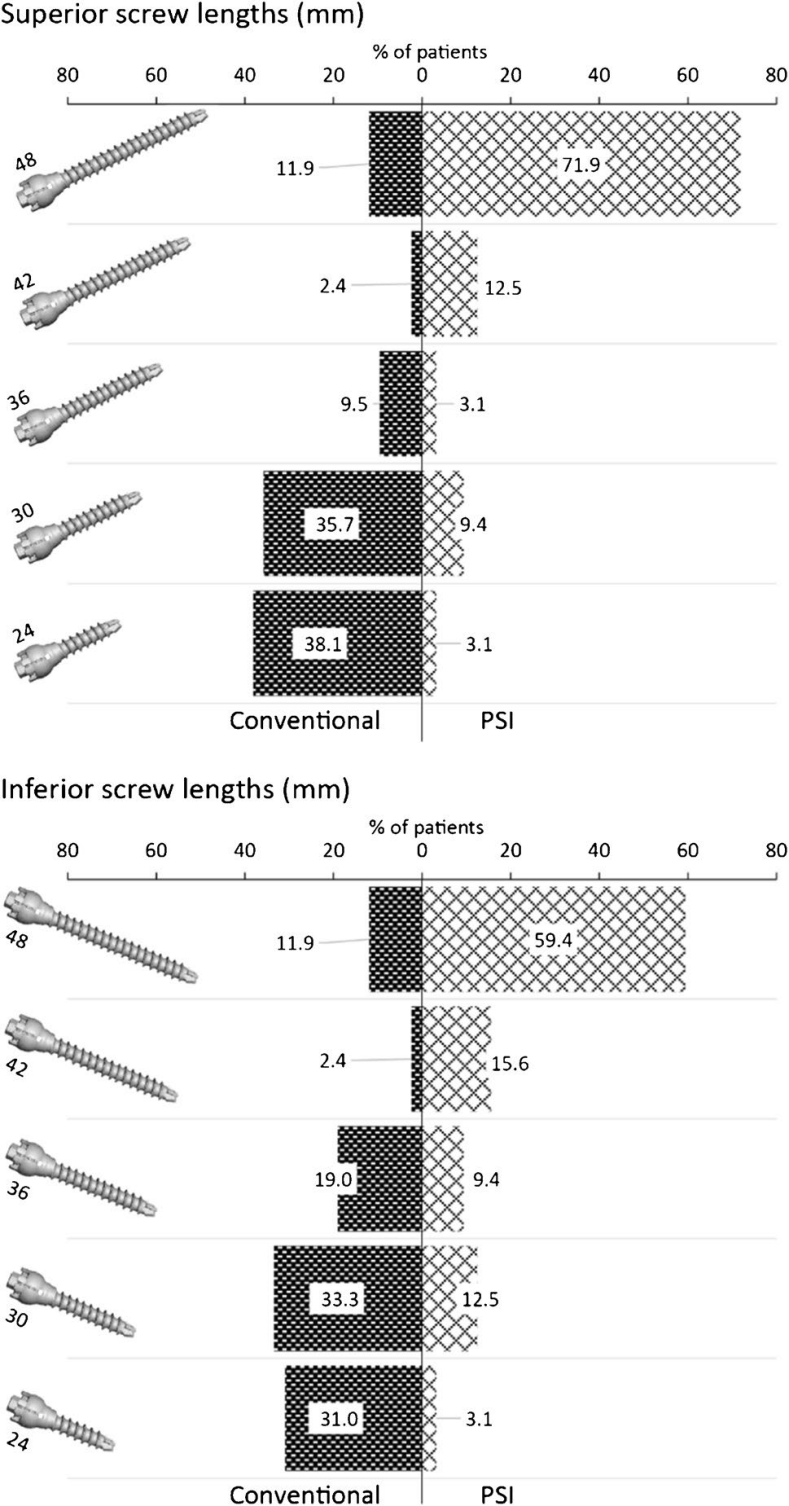
Proportion of inserted screw lengths by treatment group

Patient-specific instrumentation produced significantly longer superior (44.7 vs. 30.7 mm; *P* < 0.001) and inferior (43.0 vs. 31 mm; *P* < 0.001) screw lengths, as compared to conventional instrumentation (Table 2 and Fig. 5). A greater proportion of maximal screw lengths for the given rTSA construct (48 mm) was observed in the PSI group for both the superior and inferior screws. Operative duration was not statistically different between the PSI and CI groups (150 min vs. 169 min, respectively; *P* = 0.229), with PSI trending towards shorter operative times. None of our patients had radiological loosening of the glenoid component with an average of at least two years follow-up in the PSI group. There were, however, two cases of complications (1 PSI, 1 CI group) requiring re-operation. One patient in the PSI group suffered an early post-operative dislocation of the rTSA. Revision was performed with a thicker polyethylene liner to increase stability. One patient in the CI group had an early periprosthetic joint infection. Two debridement operations were performed to relieve the infection, and the implant was retained after a full course of antibiotics. Additionally, one patient in the PSI group had screw penetration of the inferior scapular neck discovered on post-operative X-ray due to inadequate seating of the PSI screw guide. This was not considered a complication since the screw purchase was excellent and no revision was required in the absence of neurovascular injury or impingement symptoms.

**Table 2:** Analysis of screw lengths, operative duration, and complications

Outcome	Conventional instrumentation (*n* = 42)	Patient-specific instrumentation (*n* = 31)	*P*-value
Superior screw length (mm)	30.7 ± 7.8	44.7 ± 6.7	** < 0.001**
Inferior screw length (mm)	31.7 ± 7.7	43.0 ± 7.4	** < 0.001**
Operative duration (minutes)	169 ± 50	150 ± 60	0.224
Complication incidence	1 (2.4)	1 (3.2)	1.000

Data are either mean ± SD or *n* (%). Statistically significant value *p* < 0.05

## Discussion

This study evaluated the effect of PSI for glenoid component placement on screw length, as compared to conventional instrumentation in a population of small glenoid dimensions with average of 2-year follow-up. The glenoid dimensions in our Asian population is extraordinarily small — similar to the values as reported in Korea and Japan with majority (80.8%) of glenoid diameters smaller than the manufacturer baseplate themselves [10, 11]. Difficulty arises when judging accurate placement of two 5 mm screws within an average 10.1 mm corridor scapular neck. From this study, PSI aided in screw placement with greater superior and inferior screw lengths attainable and with less variation in length. The rate of complications was similar between groups.

From the literature, glenoid component loosening occurs in 1.16% of all reverse shoulder arthroplasties and accounts for up to 13% of the indications for revision surgery [14, 15]. This is attributable to two main factors: component malpositioning and inadequate screw purchase in quality bone stock [6]. Various PSI devices have been introduced to improve glenoid baseplate positioning. The universal aim of these devices is to provide a template for accurate insertion of the central baseplate peg based on individual patient anatomy. However, there exists a wide range of guide designs, using different anatomical points of contact. Furthermore, device production may be carried out by the clinician or outsourced to a manufacturer. Many studies have reported that PSI improves glenoid component placement as compared to CI; however, the results of pooled analyses have been conflicting. While one review of 12 studies found that PSI produces lower deviations relative to pre-operative planning in version and inclination [6], another pooled analysis of 22 studies found no significant differences across these measures [16]. Such conflicting results may be explained by the fact that significant heterogeneity exists across the literature in terms of pre-operative planning, intra-operative technique, PSI construct, and postoperative evaluation employed. Fewer studies have examined the effect of PSI on screw length, with only two studies reporting that PSI improved concordance between implanted and pre-operatively planned screw length [17, 18].

Until now, no study has examined the effect of PSI on absolute screw length, which remains clinically relevant since longer screw length independently contributes to stronger glenoid baseplate fixation. Previous biomechanical studies have shown superiority of longer screw lengths with cyclical loading and baseplate displacement but further long-term clinical studies are required to determine baseplate loosening in vivo [19]. Optimal screw placement has been defined as that which maximises screw length, achieves far cortical fixation, and produces screw purchase in dense bone stock [9]. Longer screw lengths achieve stronger initial stability of the glenoid component, which may reduce the risk of glenoid loosening [19, 20]. Several authors have emphasised the importance of achieving screw purchase in dense bone beyond the glenoid vault, especially in cases with glenoid bone loss [8, 21]. Humphrey’s three-column concept identifies the coracoid base, scapular spine, and scapular pillar as regions of bone with sufficient density for screw placement [9]. These areas, however, can be difficult to locate intra-operatively due to the variation in scapular bone density across relatively short distances. Even when regions of quality bone stock are located, it remains difficult to determine the trajectory which maximises screw length in the scapular bone while reaching the far cortex. Intra-operative readjustment of a failed screw path only further reduces important bone stock. With digital preoperative planning, the surgeon is able to visualise the regions of scapular bone with the greatest density. Screw trajectories can then be optimised to pass through these regions with maximal bone purchase and then implemented accurately using patient-specific jigs.

The number of screws required for stable glenoid baseplate fixation has been a topic of recent interest. Several authors have reported achieving stable fixation, at least in the short term, using three or even two screws [9, 20, 22]. Most constructs allow for two fixed-angle locking screws in the superior and inferior positions, in addition to two non-locking screws in the anterior and posterior positions. However, recent biomechanical evidence suggests that the superior and inferior locking screws, being collinear with the direction of greatest load transmission, are of greatest importance [7]. Furthermore, the inferior screw, being nearest the applied load, appears to be the greatest contributor to fixation strength [7]. The use of compression screws may also provide early bony ingrowth to the baseplate to facilitate long-term fixation. It has been postulated that the insertion of additional screws reduces available bone stock for the more vital superior and inferior screws, thus weakening the overall construct [22]. This may be particularly impactful in populations with smaller glenoid dimensions. Our population had an average glenoid AP distance of 24.5 mm and an average scapular neck AP distance of 10.1 mm. Asian population, particularly females, have smaller glenoid dimensions relative both to Caucasians and to commercially available glenoid baseplates [10, 23, 24]. Due to the smaller scapular geometry and narrow glenoid neck, the trajectory for attaining maximal screw length becomes more difficult to determine. Digital pre-operative planning and patient-specific jigs circumvent the need to intra-operatively determine the optimal screw trajectory within this narrowed margin for error. Thus, PSI may be particularly useful in patients with smaller glenoid dimensions.

Computer navigation (CN) has been introduced as a means of improving glenoid component placement in rTSA. While initial results have been promising, CN has inherent limitations that may preclude its popularisation. In addition to the initial cost and learning curve, the potential for machine inaccuracy and extra intra-operative steps may contribute to increased surgical duration [12, 25, 26]. PSI is advantageous in that most important measurement takes place pre-operatively, and the accuracy of the guide is not subject to computer error intra-operatively. Like computer navigation, PSI requires an initial investment of learning and setup costs; however, once the learning curve has been overcome, it can be implemented at the surgeon level in a diverse range of clinical scenarios.

The PSI guide developed at our centre differs from commercially available designs in several regards. First, most commercially available PSI guides for rTSA utilise either coracoid base referencing, such as the Stryker Truesight (Stryker Orthopaedics, Mahwah, NJ, USA) and Affinis Architec PSI guide (Mathys Ltd., Bettlach, Switzerland), or rim referencing via the anterior and posterior glenoid rim, such as the Virtual Implant Positioning Glenoid Targeter (Arthrex, Naples, FL, USA) and the Signature One (Zimmer Biomet, Warsaw, IN, USA). Coracoid base referencing requires less extensive dissection but accuracy and placement may be more difficult compared to rim referencing. Our design utilises both coracoid base referencing and rim referencing to improve guide placement and accuracy. The aforementioned commercially available guides are designed for baseplate peg placement only. These are useful for situations with glenoid deformity or bone loss. However, the majority of our patients indicated for rTSA involve proximal humerus fractures without glenoid pathology. Thus, providing accurate screw trajectory for longer screw lengths to improve stability was of greater clinical importance in our population. The PSI guides from our centre consist of two components, one for the glenoid base plate and another for screw trajectories. The screw guide utilises the implant metaglene which is first scanned and rendered into a 3D object so that the guide can fit firmly onto the curvature and screw holes. Screw lengths can be predetermined with software measurements during the planning process for jig fabrication, which is analogous to computer navigation. However, these are not provided by commercially available PSI jigs.

For the clinician, there are several considerations worth noting for the use of PSI in rTSA. First, the accuracy of the PSI system is dependent on several factors, including the acquisition of an accurate 3D scapular model and ability of the surgeon to ream to the proper depth line [27]. Adequate removal of articular cartilage and soft tissue is necessary to ensure proper seating of the PSI jig and prevent deviation of the screw trajectory from the pre-operative plan. This was illustrated in our case where improper jig seating resulted in screw penetration of the scapular neck. Additionally, PSI by clinicians requires an initial input of time and expense to obtain hardware and overcome the technical learning curve necessary to produce and implement PSI guides. Despite these initial barriers, our PSI workflow in this study was carried out entirely by the investigating surgeons with a jig production time made available in as little as 24 h. The large range and average time from pre-operative CT to operation date varied due to operating theatre availability and patient medical condition changes. Once these initial investments are overcome, PSI can be feasibly conducted at the clinician level without the need for outsourcing to manufacturers.

This study had several limitations. First, no post-operative CT scans were available to confirm the final screw trajectories and analysis of final screw position. Our analysis relied on the assumption that surgeons followed the standard practice of aiming for maximal screw length in the conventional method. This was a retrospective analysis and further long-term randomised control trial should be conducted to exclude bias and co-founders for implant loosening. Furthermore, this study did not attempt to assess functional outcomes at follow-up. Long-term survivorship data including registries or large population studies are needed to determine the effect of PSI and screw length on implant loosening and longevity.

## Conclusions

In conclusion, PSI facilitates longer superior and inferior screw placement in the fixation of the glenoid component in rTSA as compared to conventional instrumentation. Furthermore, it does not appear to significantly alter operative time or complication rates. Future studies are required to compare different PSI systems and to classify different PSI guides to aid clinical practice for specific shoulder pathologies. PSI is a useful alternative to computer navigation for improving baseplate fixation in rTSA, and with sufficient training, can be implemented by surgeons themselves.

## Data Availability

Requests for data and materials are not included for personal identifiers as subjected by the centre’s Institutional Review Board ethical standards and approval.
